# The impact of the COVID-19 pandemic on liver health: exploring shifts in social and psychosocial determinants of steatosis and fibrosis

**DOI:** 10.3389/fmed.2026.1725338

**Published:** 2026-02-03

**Authors:** Yuan Zhao, Xuanhui Li, Jiacheng Cheng, Dongyu Hu, Huili Cao, Xiaojuan Wang, Junhua He, Yikun Zhu, Jin Li

**Affiliations:** 1 Department of Endocrinology and Metabolism, The Second Hospital of Shanxi Medical University, Shanxi Medical University, Taiyuan, China; 2 School of Public Health, Shanxi Medical University, Taiyuan, China; 3 Department of Cardiology, The Second Hospital of Shanxi Medical University, Shanxi Medical University, Taiyuan, China

**Keywords:** ALD, COVID-19, MASLD, MetALD, NHANES

## Abstract

**Background and objective:**

The COVID-19 pandemic profoundly disrupted social structures, psychological well-being, and lifestyle behaviors, yet the collective impact on steatotic liver disease (SLD) and fibrosis remains unclear. This study aimed to examine whether the relationships of social, psychosocial, and behavioral factors with metabolic dysfunction–associated SLD (MASLD), metabolic alcohol-related liver disease (MetALD), ALD, and liver fibrosis varied across pandemic periods.

**Methods:**

We conducted a repeated cross-sectional analysis of 6,090 adults from the National Health and Nutrition Examination Survey (NHANES) 2017–March 2020 (pre-pandemic) and 2021–2023 (pandemic), using vibration-controlled transient elastography and comprehensive social, psychosocial, and lifestyle measures. Weighted prevalence and Firth’s logistic regression assessed associations and pandemic interactions via ratios of odds ratios (RORs).

**Results:**

Age-standardized MASLD prevalence declined from 32.2% to 28.6%, especially among men, whereas advanced fibrosis nearly doubled from 2.6% to 4.2%, mainly in women. Pandemic-period interactions revealed substantial shifts in risk profiles. Higher income lost its pre-pandemic protective association with MetALD (ROR = 0.21), while unemployment increased MetALD risk in women. Depressive symptoms strengthened ALD risk in men during the pandemic (OR = 5.31; ROR = 6.73), but became less relevant for MetALD, particularly among women. Physical activity, which was previously protective for MASLD and ALD, lost significance post-pandemic. Current smoking markedly amplified ALD risk (OR rising from 3.49 to 17.08), whereas former smoking provided protection against MASLD in men. Dietary inflammation (DII) became inversely associated with fibrosis during the pandemic, and longer weekend sleep offered protective benefits for women.

**Conclusion:**

The pandemic period was associated with substantial changes in the epidemiology and psychosocial–behavioral correlates of SLD and fibrosis, with notable sex-specific differences.

## 1 Introduction

Steatotic liver disease (SLD), encompassing metabolic dysfunction-associated SLD (MASLD), metabolic alcohol-related liver disease (MetALD), and ALD, has emerged as the leading cause of chronic liver disease worldwide, with an estimated global prevalence of approximately 37.5% (1, 2). The progression of SLD is complex and multifactorial, involving insulin resistance, dysregulation of lipid metabolism, oxidative stress, inflammation, genetic predisposition, and immune system imbalances (3). These mechanisms collectively contribute to liver inflammation, fibrosis, and the potential progression to cirrhosis and cancer. However, there is an increasing recognition that social, psychological, and behavioral determinants are as critical as traditional metabolic risk factors.

Social determinants—encompassing the conditions in which people are born, live, work, and age—play a significant role in SLD outcomes (4). Studies have linked living in impoverished neighborhoods to increased liver disease severity, higher mortality, and a higher incidence of cardiovascular disease in patients with SLD (5). Additionally, health-related quality of life (HRQOL) in patients with MASLD is lower among those with obesity, physical inactivity, poor diet, and those living in disadvantaged social environments (6), highlighting the interaction between behavioral and social determinants in influencing disease severity. However, the COVID-19 pandemic has caused profound disruptions in social structures, psychological health, and lifestyle behaviors worldwide. Pandemic-related lockdowns, social isolation, and economic instability triggered significant changes in lifestyle, including physical inactivity, poor diet, sleep disturbances, increased stress, and heightened tobacco and alcohol use—factors all associated with higher risks of chronic illness and the exacerbation of pre-existing conditions (7, 8). As a central organ in metabolic homeostasis and a target of alcohol-induced injury, the liver is particularly vulnerable to these psychosocial and behavioral disruptions.

While prior studies have primarily focused on the isolated impact of COVID-19 on the prevalence of SLD and fibrosis, they often relied on small samples, lacked national representativeness, and seldom considered the interplay of structural, psychosocial, and behavioral factors (9–11). Moreover, whether the patterns of association between these factors and liver disease outcomes differed across pandemic periods, and how such patterns varied among MASLD, MetALD, ALD, and fibrosis risk, remain insufficiently characterized. Using nationally representative data from the National Health and Nutrition Examination Survey (NHANES) from 2017 to 2023, this study aimed to examine associations between social, psychosocial, and lifestyle factors and liver disease phenotypes before and during the COVID-19 pandemic, with particular attention to sex-specific and pandemic-period interactions.

## 2 Methods

### 2.1 Study population

This study is a repeated cross-sectional analysis of NHANES survey cycles conducted before (2017–March 2020) and during (2021–2023) the COVID-19 pandemic. NHANES applies a multistage, stratified, probability sampling design to yield nationally representative estimates of the non-institutionalized US population (12). Adults aged ≥20 years with available liver elastography, alcohol consumption, metabolic, social, psychosocial, and lifestyle data were included. Participants were excluded if they had evidence of viral hepatitis or lacked information on essential variables required for exposure, outcome, or covariate definition. These variables comprised indicators of glycemic status (fasting plasma glucose or hemoglobin A1c), key sociodemographic characteristics (age, sex, race/ethnicity, educational attainment, family income-to-poverty ratio, marital status, employment status, health insurance coverage, and country of birth), psychosocial assessment of depressive symptoms using the Patient Health Questionnaire-9, and major lifestyle-related factors, including smoking status, alcohol intake, physical activity, sleep duration, and sedentary behavior. The final sample comprised 6,090 participants (3,621 pre-pandemic; 2,469 pandemic) (Supplementary Figure S1).

### 2.2 Clinical and laboratory evaluations

#### 2.2.1 Sociodemographic characteristics

Information on sociodemographic characteristics was obtained through standardized NHANES questionnaires administered by trained interviewers. Variables included age (years) and sex (men or women). Race and ethnicity were categorized as non-Hispanic White, non-Hispanic Black, Mexican American, other Hispanic, non-Hispanic Asian, and other races. Socioeconomic position was assessed using educational attainment (less than college, some college, or college degree or above) and the family poverty–income ratio (PIR), which was classified as low (<1.30), middle (1.30–3.50), or high (≥3.50). Additional social indicators included marital status (never married, divorced/separated/widowed, or married/living with a partner), employment status (not employed, part-time, or full-time), health insurance coverage (insured or uninsured), and place of birth (US-born or foreign-born).

#### 2.2.2 Psychological factors

Psychological status was evaluated using the Patient Health Questionnaire-9 (PHQ-9), a validated screening tool for depressive symptoms. A total PHQ-9 score of 10 or higher was used to identify individuals with moderate-to-severe depressive symptoms, consistent with established clinical and epidemiological practice (13).

#### 2.2.3 Lifestyle and behavioral factors

Health-related behaviors were assessed using self-reported questionnaire data. Smoking status was classified as never, former, or current smoker. Alcohol intake was quantified as grams of ethanol consumed per week and analyzed as a continuous exposure. Sleep duration was derived from reported habitual sleep hours on both weekdays and weekends and categorized as short (<6 h/night), normal (6–8 h/night), or long (≥8 h/night). Physical activity was calculated based on reported frequency, duration, and intensity of leisure-time activities performed during the preceding 7 days. Following World Health Organization 2020 guidelines (14), total weekly physical activity was computed as the sum of moderate-intensity minutes plus twice the minutes of vigorous-intensity activity and categorized as low (<150 min/week), moderate (150–300 min/week), or high (≥300 min/week). Sedentary behavior was assessed as the self-reported average number of hours spent sitting per day.

#### 2.2.4 Dietary assessment

Dietary intake was evaluated using 24-h dietary recall interviews conducted by NHANES. Intakes of total energy, macronutrients, dietary fiber, fats (including saturated and polyunsaturated fatty acids), cholesterol, selected vitamins, minerals, and other micronutrients were estimated using the USDA Food and Nutrient Database for Dietary Studies. Two summary dietary indices were derived. The Dietary Inflammatory Index (DII) was calculated according to the published scoring algorithm, in which individual nutrient intakes were standardized to a global reference database, transformed into centered percentiles, weighted by literature-derived inflammatory effect scores, and summed to generate an overall index reflecting the inflammatory potential of the diet (15). The Composite Dietary Antioxidant Index (CDAI) was constructed by standardizing intakes of six antioxidant-related nutrients (vitamins A, C, and E, selenium, zinc, and manganese) and summing the resulting Z-scores to reflect cumulative dietary antioxidant capacity (16). As no validated clinical cut-points exist for either index, both DII and CDAI were treated as continuous variables in the analyses.

#### 2.2.5 Clinical measurements

Standardized physical examinations and laboratory assessments were conducted in NHANES mobile examination centers. Anthropometric and clinical data included height, weight, waist circumference (WC), and blood pressure. Laboratory measures included fasting plasma glucose (FPG), hemoglobin A1c (HbA1c), total cholesterol (TC) and high-density lipoprotein cholesterol (HDL-C).

Vibration-controlled transient elastography (VCTE) measurements were performed using identical FibroScan® 502 Touch devices, operated by NHANES-certified technicians. Standardized protocols were followed across all study cycles, including specific patient positioning, and a requirement of ≥10 valid measurements with an interquartile range to median ratio of <30%. NHANES enforces strict cross-cycle calibration procedures to ensure the comparability of Controlled Attenuation Parameter (CAP) and Liver Stiffness Measurement (LSM) values between survey periods.

### 2.3 Definitions

Cardiometabolic risk factors (CMRFs) were defined by standard clinical thresholds. Overweight/obesity was identified by body mass index (BMI) ≥ 25 kg/m^2^ (≥23 kg/m^2^ for Asians) or WC ≥ 94 cm (men) / ≥80 cm (women). Diabetes was defined by self-report, glucose-lowering medication use, FPG ≥ 126 mg/dL, or HbA1c ≥ 6.5%; prediabetes as fasting glucose 100–125 mg/dL or HbA1c 5.7–6.4% without diabetes. Hypertension was defined as BP ≥ 130/85 mmHg or antihypertensive treatment. Dyslipidemia was defined as triglycerides ≥1.70 mmol/L or lipid-lowering therapy; low HDL-C as ≤1.0 mmol/L in men or ≤1.3 mmol/L in women.

SLD was defined by a CAP ≥ 285 dB/m, a threshold validated against liver biopsy and widely applied in epidemiologic and NHANES-based studies to identify the presence of hepatic steatosis (17). The SLD phenotypes were classified as follows (18):

MASLD: Hepatic steatosis in the presence of ≥1 cardiometabolic risk factor (CMRF) without significant alcohol consumption.

MetALD: Hepatic steatosis with ≥1 CMRF and moderate alcohol consumption (140–350 g/week for women; 210–420 g/week for men).

ALD: Hepatic steatosis with heavy alcohol use (>350 g/week for women; >420 g/week for men), regardless of CMRFs, or alcohol intake exceeding moderate thresholds in the absence of CMRFs (>140 g/week for women; >210 g/week for men).

Cryptogenic SLD: Hepatic steatosis with no identifiable metabolic or alcohol-related etiology.

Alcohol Intake Calculation: Weekly alcohol consumption was derived from NHANES alcohol questionnaire items (ALQ121 and ALQ130). Annual drinking days were estimated from reported frequency, and drinks per drinking day were multiplied by 14 g of pure alcohol. Weekly intake was then calculated as: Weekly intake (g/week) = (Annual drinking days / 365) × 7 × Drinks per day × 14.

Liver fibrosis was staged using LSM thresholds of ≥8.2 kPa for significant fibrosis (≥F2), ≥9.7 kPa for advanced fibrosis (≥F3), and ≥13.6 kPa for cirrhosis (F4), based on biopsy-validated cutoffs commonly used in population-based studies employing VCTE (19).

### 2.4 Statistical analysis

All analyzes incorporated NHANES sampling weights, strata, and clusters to ensure national representativeness. Continuous variables were summarized as weighted medians (interquartile range), and categorical variables as counts (weighted percentages). Age-standardized prevalences for MASLD, MetALD, ALD, and fibrosis stages were estimated via direct standardization to the 2,000 US Census population.

Given the relatively low prevalence of MetALD and ALD, associations between determinants and liver outcomes were examined using Firth’s penalized-likelihood logistic regression to reduce small-sample bias. All models were adjusted for potential confounders, including age, race, hypertension, diabetes mellitus, and BMI. Interaction effects were examined using pre-specified two-way and three-way interaction terms, with a primary focus on effect modification by pandemic period and sex. To quantify interaction on the multiplicative scale, ratios of odds ratios (RORs) were calculated by exponentiating the corresponding interaction coefficients. Consistent with hypothesis-driven analyses of effect modification, interpretation emphasized the direction, magnitude, and consistency of interaction effects rather than isolated statistical significance of individual subgroup estimates. Statistical significance was defined as a two-sided *p* < 0.05. Analyzes were performed in R (version 4.5.1) using the survey and nhanesR packages.

## 3 Results

### 3.1 Baseline characteristics and pandemic-associated shifts in social and psychosocial determinants

A total of 6,090 adults were included in the study, with 3,621 surveyed pre-COVID-19 (2019–2020) and 2,469 post-COVID-19 (2021–2023). The median age was 46.0 years, with individuals with SLD being significantly older than those without SLD (Table 1). Post-pandemic, the cohort had a higher proportion of unemployed individuals (37.5% vs. 21.2% in MASLD) and a higher prevalence of depressive symptoms (32.8% vs. 23.2% in MASLD). Obesity and hypertension remained prevalent across all liver disease groups. Median CAP and LSM values were higher in the MASLD, MetALD, and ALD groups compared to the No SLD group, indicating more severe steatosis and fibrosis (Table 2). Post-pandemic, lifestyle patterns shifted, including a decrease in the proportion of “low active” individuals (48.8% vs. 64.9% in MASLD), modest reductions in energy and carbohydrate intake, and increased weekday sleep duration.

**Table 1:** Sociodemographic and clinical characteristics of the study population by steatotic liver disease.

Variables	Total (*n* = 6,090)	2017–2020 (*n* = 3,621)				2021–2023 (*n* = 2,469)				*p*-value
		No SLD (*n* = 2,298)	MASLD (*n* = 1,204)	MetALD (*n* = 89)	ALD (*n* = 30)	No SLD (*n* = 1,669)	MASLD (*n* = 714)	MetALD (*n* = 65)	ALD (*n* = 21)	
Age years	46.0 (33.0, 60.0)	42.0 (29.0, 57.0)	50.0 (36.0, 61.0)	53.0 (41.0, 63.0)	53.0 (37.0, 57.0)	46.0 (33.0, 62.0)	51.0 (38.0, 63.0)	50.0 (39.0, 66.0)	51.0 (50.0, 60.0)	<0.0001
Sex										<0.0001
Men	2,916 (48.9)	1,056 (44.1)	677 (56.5)	59 (62.4)	26 (92.4)	696 (45.9)	348 (53.3)	37 (61.7)	14 (76.4)	
Women	3,174 (51.1)	1,242 (55.9)	527 (43.5)	30 (37.6)	4 (7.6)	970 (54.1)	366 (46.7)	28 (38.3)	7 (23.6)	
Race										0.1
Non-Hispanic White	3,102 (68.6)	886 (72.0)	483 (70.6)	51 (82.4)	10 (52.2)	1,113 (70.3)	488 (74.0)	52 (82.6)	18 (93.0)	
Non-Hispanic Black	1,157 (9.6)	650 (11.5)	249 (8.0)	17 (7.2)	5 (4.3)	179 (11.6)	55 (6.6)	2 (3.6)	0 (0.0)	
Mexican American	521 (6.3)	181 (5.3)	188 (11.1)	12 (7.1)	10 (28.6)	74 (4.9)	51 (7.6)	4 (6.1)	1 (2.8)	
Non-Hispanic Asian	432 (4.1)	236 (4.4)	102 (3.7)	2 (0.9)	1 (3.1)	63 (5.0)	27 (4.2)	0 (0.0)	0 (0.0)	
Married status										<0.001
Never married	1,241 (19.5)	551 (23.6)	178 (13.5)	14 (11.3)	5 (10.5)	363 (21.7)	112 (13.2)	11 (18.1)	6 (18.9)	
Divorced, separated or widowed	1,263 (16.2)	457 (16.9)	233 (15.0)	13 (11.5)	6 (16.7)	365 (15.2)	168 (18.3)	16 (21.5)	5 (27.5)	
Married or living with partner	3,586 (64.2)	1,290 (59.5)	793 (71.6)	62 (77.3)	19 (72.8)	938 (63.1)	434 (68.5)	38 (60.3)	10 (53.5)	
Place of birth										0.03
US-born	4,890 (85.9)	1784 (85.9)	903 (84.4)	79 (93.6)	19 (66.6)	1,403 (85.1)	619 (87.9)	61 (96.3)	20 (97.3)	
Born outside the US	1,200 (14.1)	514 (14.1)	301 (15.6)	10 (6.4)	11 (33.4)	263 (14.9)	95 (12.1)	4 (3.7)	1 (2.7)	
Educational levels										<0.001
Less than college	1,517 (24.9)	623 (24.6)	386 (33.4)	25 (27.2)	16 (42.1)	297 (19.7)	144 (24.0)	19 (39.4)	6 (29.9)	
Some college	2,260 (34.0)	900 (33.2)	499 (37.0)	39 (41.8)	12 (54.8)	506 (30.3)	273 (39.0)	23 (33.6)	8 (49.3)	
College graduate or above	2,313 (41.1)	775 (42.2)	319 (29.5)	25 (30.9)	2 (3.1)	863 (50.0)	297 (37.0)	23 (27.0)	7 (20.8)	
PIR levels										0.05
Low income	1,129 (13.1)	529 (15.1)	245 (12.2)	9 (4.4)	10 (22.7)	239 (13.0)	84 (10.0)	9 (16.1)	4 (13.6)	
Middle income	2,179 (32.7)	824 (29.8)	476 (35.1)	36 (28.7)	15 (38.1)	531 (32.1)	272 (39.7)	17 (28.6)	7 (37.0)	
High income	2,782 (54.1)	945 (55.1)	483 (52.7)	44 (66.9)	5 (39.1)	896 (54.9)	358 (50.3)	39 (55.3)	10 (49.4)	
Health insurance	5,403 (91.1)	1939 (87.9)	1,039 (90.5)	75 (91.1)	18 (70.4)	1,578 (94.5)	672 (93.3)	63 (96.3)	16 (77.4)	<0.0001
Private insurance	3,672 (67.9)	1,301 (67.2)	713 (68.4)	60 (81.8)	10 (45.2)	1,076 (68.0)	457 (68.1)	42 (66.4)	11 (51.4)	0.3
Smoking status										<0.0001
Never	3,617 (60.5)	1,390 (60.1)	682 (58.5)	26 (23.5)	11 (37.9)	1,044 (65.5)	432 (61.3)	25 (39.5)	5 (14.9)	
Ex-smoker	1,518 (25.3)	486 (23.1)	340 (30.1)	27 (40.2)	8 (30.2)	433 (23.2)	189 (25.8)	25 (37.6)	9 (40.2)	
Current smoker	955 (14.3)	422 (16.8)	182 (11.4)	36 (36.2)	11 (31.9)	189 (11.4)	93 (13.0)	15 (22.9)	7 (45.0)	
Work										0.02
Not employed	2,395 (33.6)	815 (29.7)	437 (31.2)	29 (32.1)	12 (40.2)	736 (37.7)	322 (37.5)	34 (42.5)	9 (41.8)	
Part-time employee	855 (14.2)	370 (17.0)	166 (13.7)	9 (12.2)	6 (12.3)	222 (13.2)	72 (10.3)	5 (7.7)	5 (20.8)	
Full-time employee	2,840 (52.1)	1,113 (53.3)	601 (55.1)	51 (55.7)	12 (47.5)	708 (49.0)	320 (52.2)	26 (49.8)	7 (37.4)	
Prediabetes	432 (6.6)	116 (4.9)	120 (11.1)	15 (13.6)	7 (15.3)	89 (4.4)	72 (8.4)	11 (14.8)	2 (6.7)	<0.0001
T2D	895 (11.7)	210(5.5)	358 (25.4)	14 (12.7)	5 (19.7)	117(5.9)	182 (22.8)	5(7.5)	4 (28.1)	<0.0001
Obesity	2,533 (40.7)	642 (26.0)	854 (72.7)	52 (55.8)	20 (69.0)	404 (23.7)	509 (72.1)	37 (54.7)	15 (67.6)	<0.0001
Hypertension	2,499 (34.9)	785 (25.7)	654 (52.4)	50 (48.8)	17 (60.9)	577 (29.0)	364 (44.5)	40 (60.4)	12 (63.7)	<0.0001
CVD	521 (6.7)	174 (4.9)	125 (9.9)	8 (10.1)	3 (14.7)	135 (5.8)	68 (8.2)	6(7.5)	2 (17.6)	0.003
Depression levels										<0.0001
No/minimal depression	4,516 (74.9)	1787 (79.1)	903 (76.8)	61 (64.2)	22 (79.5)	1,203 (73.2)	480 (67.2)	48 (72.2)	10 (37.2)	
Depression symptoms	1,574 (25.1)	511 (20.9)	301 (23.2)	28 (35.8)	8 (20.5)	463 (26.8)	234 (32.8)	17 (27.8)	11 (62.8)	

Data are presented as weighted medians (interquartile ranges) for continuous variables and percentages for categorical variables. MASLD, metabolic dysfunction-associated steatotic liver disease; MetALD, metabolic alcohol-related liver disease; ALD, alcohol-associated liver disease; PIR, poverty income ratio; T2D, type 2 diabetes mellitus; CVD, cardiovascular disease.

**Table 2:** Biochemical parameters and lifestyle behaviors of the study population stratified by steatotic liver disease phenotypes.

Variables	Total (*n* = 6,090)	2017–2020 (*n* = 3,621)				2021–2023 (*n* = 2,469)				*p*-value
		No SLD (*n* = 2,298)	MASLD (*n* = 1,204)	MetALD (*n* = 89)	ALD (*n* = 30)	No SLD (*n* = 1,666)	MASLD (*n* = 714)	MetALD (*n* = 65)	ALD (*n* = 21)	
BMI, kg/m^2^	28.3 (24.6, 33.1)	26.4 (23.3, 30.1)	33.8 (29.6, 38.2)	31.3 (26.2, 33.0)	33.4 (29.7, 36.8)	26.1 (23.6, 29.6)	33.5 (29.6, 38.4)	31.0 (27.8, 35.4)	32.5 (28.6, 34.7)	<0.0001
WC, cm	98.6 (88.2, 110.4)	92.7 (83.0, 102.0)	112.1 (102.5, 121.9)	105.8 (98.3, 116.0)	115.8 (107.1, 122.9)	92.8 (84.0, 101.3)	111.5 (102.4, 121.8)	106.9 (100.6, 115.1)	112.5 (99.5, 123.5)	<0.0001
WHR	0.9 (0.9, 1.0)	0.9 (0.8, 1.0)	1.0 (0.9, 1.0)	1.0 (0.9, 1.0)	1.0 (1.0, 1.1)	0.9 (0.8, 1.0)	1.0 (0.9, 1.0)	1.0 (0.9, 1.0)	1.0 (1.0, 1.1)	<0.0001
WHtR	0.6 (0.5, 0.7)	0.6 (0.5, 0.6)	0.7 (0.6, 0.7)	0.6 (0.6, 0.7)	0.7 (0.6, 0.7)	0.5 (0.5, 0.6)	0.7 (0.6, 0.7)	0.6 (0.6, 0.7)	0.7 (0.6, 0.7)	<0.0001
FPG, mmol/L	5.6 (5.2, 6.1)	5.5 (5.2, 5.8)	5.9 (5.6, 6.5)	5.6 (5.3, 6.3)	6.2 (5.8, 6.3)	5.4 (5.1, 5.8)	5.8 (5.4, 6.6)	5.7 (5.5, 6.4)	7.4 (5.6, 11.2)	<0.0001
HbA1c,%	5.4 (5.2, 5.7)	5.3 (5.1, 5.6)	5.6 (5.3, 6.1)	5.4 (5.2, 5.7)	5.7 (5.3, 5.8)	5.4 (5.1, 5.6)	5.6 (5.3, 6.1)	5.4 (5.1, 5.6)	5.8 (5.2, 6.4)	<0.0001
SBP	118.7 (109.3, 129.0)	115.7 (107.7, 126.0)	121.7 (112.0, 131.7)	127.3 (120.0, 136.7)	130.3 (121.3, 139.3)	117.3 (108.7, 128.7)	121.3 (112.0, 131.0)	130.0 (121.3, 140.0)	126.7 (120.7, 133.0)	<0.0001
DBP	74.0 (67.7, 81.0)	71.3 (65.3, 78.0)	77.0 (69.7, 84.0)	80.3 (74.3, 86.0)	80.3 (74.3, 88.7)	73.3 (67.0, 80.0)	76.7 (71.0, 84.3)	81.3 (75.0, 89.0)	79.3 (76.7, 86.7)	<0.0001
TC, mmol/L	4.9 (4.2, 5.6)	4.7 (4.2, 5.5)	4.9 (4.2, 5.7)	5.2 (4.9, 5.9)	5.7 (5.0, 6.3)	4.9 (4.3, 5.5)	4.9 (4.2, 5.6)	5.5 (4.3, 6.1)	5.4 (4.9, 6.9)	<0.0001
HDL-C, mmol/L	1.4 (1.1, 1.7)	1.5 (1.2, 1.7)	1.2 (1.0, 1.4)	1.5 (1.2, 1.7)	1.3 (1.1, 1.7)	1.5 (1.2, 1.7)	1.2 (1.1, 1.4)	1.3 (1.2, 1.6)	1.2 (0.9, 1.6)	<0.0001
CAP	257.0 (216.0, 304.0)	227.0 (199.0, 256.0)	327.0 (303.0, 355.0)	321.0 (293.0, 363.0)	347.0 (323.0, 355.0)	231.0 (200.0, 255.0)	323.0 (303.0, 358.0)	320.0 (304.0, 356.0)	358.0 (335.0, 379.0)	<0.0001
LSM	4.9 (4.0, 6.1)	4.5 (3.8, 5.5)	5.5 (4.5, 7.2)	5.4 (4.6, 6.7)	6.3 (5.0, 8.3)	4.6 (3.8, 5.6)	5.9 (4.9, 7.6)	6.1 (5.0, 8.3)	6.9 (6.1, 12.8)	<0.0001
Dietary composition										
Energy intake, kcal	1,990.0 (1,496.0, 2,641.0)	1,996.0 (1,489.0, 2,591.0)	2,158.0 (1,595.0, 2,833.0)	2,513.0 (1,857.0, 3,209.0)	2,685.0 (2,311.0, 2,814.0)	1,925.0 (1,431.0, 2,510.0)	1,958.0 (1,474.0, 2,566.0)	2,167.0 (1,436.0, 2,877.0)	1,989.0 (1,272.0, 2,894.0)	<0.0001
Carbohydrates, g	218.3 (157.2, 297.2)	220.2 (155.4, 292.7)	239.2 (170.2, 325.9)	219.9 (143.5, 310.9)	220.0 (200.2, 312.3)	207.0 (148.7, 289.4)	214.0 (158.3, 281.7)	201.4 (152.9, 289.6)	190.5 (144.7, 247.3)	<0.0001
Sugars, g	84.2 (51.8, 128.2)	84.2 (49.8, 127.6)	96.6 (60.3, 141.9)	67.8 (55.7, 111.3)	57.4 (38.3, 128.4)	80.0 (50.0, 120.1)	86.3 (53.5, 128.1)	69.4 (29.3, 128.0)	72.1 (49.1, 91.4)	<0.0001
Dietary fiber, g	14.5 (9.6, 20.7)	14.5 (9.6, 20.8)	14.3 (9.2, 20.1)	15.0 (8.7, 19.8)	15.8 (8.7, 19.0)	15.0 (10.3, 21.8)	13.7 (9.4, 20.4)	13.0 (7.1, 19.7)	10.9 (9.0, 17.2)	<0.0001
Proteins, g	73.9 (53.8, 100.6)	73.1 (53.8, 98.8)	80.7 (58.1, 107.7)	84.8 (61.9, 122.8)	79.2 (67.9, 92.4)	71.0 (52.1, 98.8)	71.2 (50.3, 97.0)	73.2 (54.2, 96.4)	70.5 (40.0, 93.6)	0.001
Fats, g	80.9 (57.3, 113.5)	79.7 (56.6, 112.9)	87.7 (62.2, 122.4)	98.2 (55.6, 126.5)	85.9 (66.9, 105.6)	78.6 (55.8, 109.8)	80.3 (57.9, 112.2)	90.4 (54.1, 111.6)	70.4 (42.8, 85.0)	0.003
Saturated fats, g	25.8 (17.0, 37.5)	26.0 (16.8, 37.4)	28.4 (19.5, 41.5)	33.1 (20.4, 42.7)	28.5 (20.8, 34.1)	24.3 (15.9, 35.7)	26.6 (18.0, 36.8)	25.6 (15.1, 36.3)	23.1 (12.3, 25.1)	<0.001
Polyunsaturated fats, g	18.1 (11.9, 26.7)	17.5 (11.4, 26.8)	19.1 (12.9, 27.5)	21.5 (13.3, 29.3)	22.0 (12.5, 28.5)	18.0 (11.8, 25.8)	17.9 (11.9, 27.3)	19.7(9.8, 25.8)	12.5 (10.8, 21.4)	0.04
Cholesterol, mg	250.0 (140.0, 433.0)	239.0 (130.0, 427.0)	291.0 (161.0, 454.0)	301.0 (168.0, 435.0)	325.0 (243.0, 578.0)	230.0 (132.0, 416.0)	238.0 (135.0, 437.0)	319.0 (168.0, 534.0)	220.0 (164.0, 479.0)	<0.0001
Linoleic acid, g	16.0 (10.3, 23.8)	15.5 (10.0, 23.9)	17.0 (11.3, 24.4)	19.3 (12.1, 25.6)	19.8 (11.6, 25.5)	15.9 (10.2, 22.7)	16.1 (10.2, 24.2)	17.1 (8.9, 22.9)	10.9 (9.3, 19.4)	0.03
Vitamin D, μg	3.0 (1.2, 5.6)	2.9 (1.1, 5.2)	3.1 (1.2, 5.7)	3.0 (1.7, 4.1)	3.5 (2.2, 5.0)	3.1 (1.2, 6.1)	2.9 (1.2, 5.5)	3.4 (1.5, 5.8)	3.4 (1.2, 4.7)	0.03
Vitamin E, mg	8.1 (5.3, 12.1)	8.0 (5.2, 11.9)	8.2 (5.3, 11.7)	8.3 (5.6, 11.7)	8.1 (5.5, 11.0)	8.3 (5.4, 12.6)	7.8 (5.0, 11.9)	8.2 (5.2, 11.6)	6.6 (4.4, 9.1)	0.2
Vitamin K, μg	79.9 (44.7, 147.8)	79.6 (43.8, 154.2)	75.6 (43.7, 135.1)	99.8 (53.3, 173.0)	76.6 (44.1, 146.1)	84.1 (46.9, 154.8)	79.2 (44.5, 131.3)	74.1 (46.5, 149.8)	69.7 (55.3, 97.1)	0.3
Sodium, mg	3,115.0 (,2272.0, 4,226.0)	3,170.0 (2,310.0, 4,206.0)	3,454.0 (2,540.0, 4,636.0)	3,765.0 (,2749.0, 4,987.0)	3,336.0 (2,501.0, 40,83.0)	2,859.0 (2,126.0, 3,904.0)	3,051.0 (2,214.0, 4,261.0)	2,980.0 (2,250.0, 4,550.0)	2,917.0 (2,316.0, 4,413.0)	< 0.0001
Magnesium, mg	280.0 (204.0, 375.0)	279.0 (207.0, 378.0)	282.0 (206.0, 376.0)	319.0 (208.0, 440.0)	359.0 (276.0, 378.0)	284.0 (204.0, 381.0)	262.0 (193.0, 351.0)	257.0 (183.0, 354.0)	292.0 (206.0, 346.0)	0.03
Selenium, μg	102.6 (71.6, 140.4)	100.1 (70.2, 139.9)	110.9 (80.8, 149.9)	121.7 (89.4, 165.7)	127.1 (95.6, 160.3)	98.8 (67.9, 134.5)	99.2 (69.3, 134.1)	97.6 (69.9, 150.4)	98.9 (53.4, 150.0)	<0.001
Caffeine, mg	138.0 (44.0, 243.0)	144.0 (43.0, 248.0)	144.0 (49.0, 257.0)	192.0 (47.0, 292.0)	158.0 (80.0, 351.0)	123.0 (42.0, 217.0)	135.0 (42.0, 240.0)	144.0 (45.0, 240.0)	126.0 (46.0, 314.0)	0.1
CDAI	−0.2 (−0.6, 0.4)	−0.1 (−0.6, 0.5)	−0.1 (−0.6, 0.5)	0.1 (−0.7, 0.8)	−0.1 (−0.6, 0.2)	−0.2 (−0.6, 0.4)	−0.2 (−0.7, 0.3)	−0.3 (−0.9, 0.3)	−0.3 (−1.0, 0.1)	0.2
DII	0.1 (−0.7, 0.8)	0.1 (−0.7, 0.8)	0.1 (−0.7, 0.8)	−0.2 (−1.0, 0.6)	−0.1 (−1.0, 0.3)	0.1 (−0.8, 0.8)	0.2 (−0.4, 0.8)	0.2 (−0.6, 0.9)	0.2 (−0.2, 0.7)	0.1
Alcohol consumption, g/week	16.1 (3.2, 56.4)	16.1 (3.2, 55.8)	8.1 (2.4, 40.3)	293.2 (196.0, 322.2)	586.4 (483.3, 784.0)	24.2 (3.6, 80.5)	12.1 (2.4, 48.9)	244.3 (241.6, 322.2)	588.0 (483.3, 644.4)	<0.0001
PA levels										<0.0001
Low active	3,209 (48.7)	1,274 (49.9)	816 (64.9)	57 (62.6)	25 (86.3)	635 (36.9)	364 (48.8)	27 (40.3)	10 (44.7)	
Moderate active	937 (16.7)	283 (13.8)	147 (14.5)	10 (11.1)	4 (11.6)	334 (20.1)	142 (20.2)	14 (22.0)	2(6.5)	
High active	1,944 (34.6)	741 (36.3)	241 (20.6)	22 (26.3)	1(2.1)	697 (43.0)	208 (31.0)	24 (37.6)	9 (48.8)	
Sleep hours (week days)										<0.001
<6 h	543 (7.2)	226 (7.5)	135 (8.8)	5 (2.3)	5 (10.9)	110 (6.5)	55 (6.7)	6 (7.1)	1 (2.7)	
6–8 h	2,658 (45.3)	1,003 (44.2)	587 (54.3)	38 (44.9)	13 (62.1)	679 (41.4)	305 (45.8)	26 (40.7)	6 (40.1)	
≥8 h	2,889 (47.4)	1,069 (48.3)	482 (36.9)	46 (52.8)	12 (27.0)	877 (52.2)	354 (47.6)	33 (52.3)	14 (57.2)	
Sleep hours (weekends)										0.5
<6 h	327 (4.1)	136 (4.1)	79 (5.5)	5 (2.4)	4 (9.9)	65 (3.5)	34 (4.0)	3 (4.0)	1 (2.9)	
6–8 h	1,631 (25.9)	629 (26.0)	330 (27.8)	22 (24.3)	3 (15.7)	432 (24.8)	190 (26.9)	21 (33.8)	3 (10.2)	
≥8 h	4,102 (69.6)	1,520 (70.0)	791 (66.7)	61 (73.3)	23 (74.3)	1,163 (71.7)	486 (69.1)	40 (62.2)	16 (86.9)	

Data are presented as weighted medians (interquartile ranges) for continuous variables and percentages for categorical variables. MASLD, metabolic dysfunction-associated steatotic liver disease; MetALD, metabolic alcohol-related liver disease; ALD, alcohol-associated liver disease; BMI, body mass index; WC, waist circumference; WHR, waist-to-hip ratio; WHtR, waist-to-height ratio; FPG, fasting plasma glucose; SBP, systolic blood pressure; DBP, diastolic blood pressure; TC, total cholesterol; HDL-C, high-density lipoprotein cholesterol; CAP, controlled attenuation parameter; LSM, liver stiffness measurement; CDAI, composite dietary antioxidant index; DII, dietary inflammatory index; PA, physical activity.

### 3.2 Prevalence of SLD and fibrosis stages pre- and post-COVID-19 pandemic

**Figure 1:**
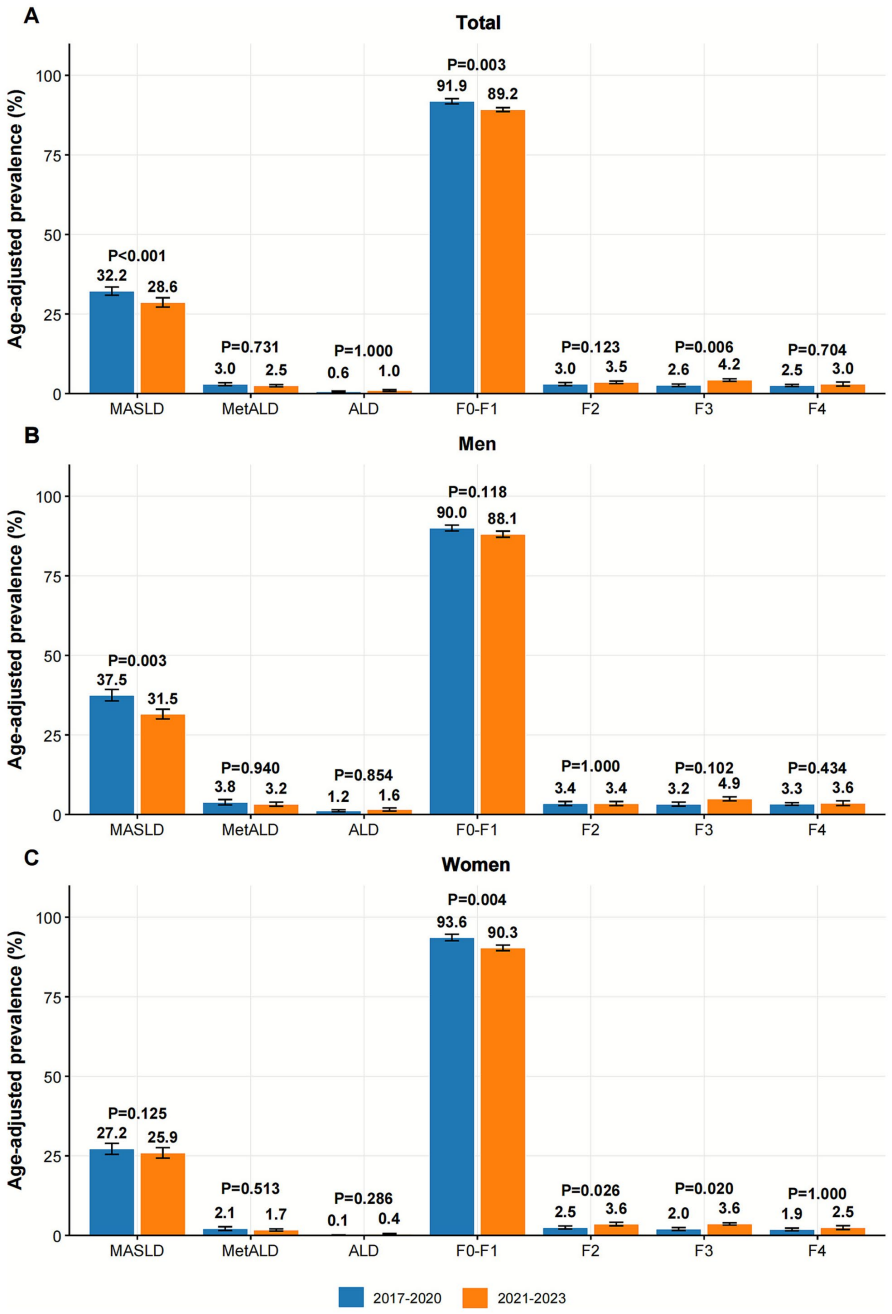
Age-adjusted prevalence of MASLD, MetALD, ALD, and liver fibrosis stages pre- and post-COVID-19 pandemic. **(A)** Age-adjusted prevalence of MASLD, MetALD, ALD, and liver fibrosis stages before and after the COVID-19 pandemic in the total population. **(B)** Age-adjusted prevalence of MASLD, MetALD, ALD, and liver fibrosis stages before and after the COVID-19 pandemic in men participants. **(C)** Age-adjusted prevalence of MASLD, MetALD, ALD, and liver fibrosis stages before and after COVID-19 pandemic in women participants. MASLD, Metabolic dysfunction-associated steatotic liver disease; MetALD, metabolic alcohol-related liver disease; ALD, alcohol-associated liver disease; F0, no fibrosis; F1, mild fibrosis; F2, moderate fibrosis; F3, severe fibrosis; F4, cirrhosis.

The analysis of age-adjusted prevalence rates revealed significant changes in the burden of SLD and fibrosis following the COVID-19 pandemic. Overall, MASLD prevalence decreased significantly from 32.2% to 28.6% post-pandemic, primarily driven by men (37.5% to 31.5%), while changes among women were not significant (Figure 1). The prevalence of MetALD and ALD remained stable. The proportion of individuals with no fibrosis or minimal fibrosis (F0–F1) declined from 91.9% to 89.2%, while the prevalence of severe fibrosis (F3) increased from 2.6% to 4.2%. Changes in moderate fibrosis (F2) and cirrhosis (F4) were not significant. Sex-stratified analysis revealed that women experienced notable increases in the prevalence of both stage F2 (2.5% to 3.6%) and stage F3 fibrosis (2.0% to 3.6%), while men maintained a higher prevalence of F4 in both periods.

### 3.3 Associations and interactions of social and psychosocial determinants with SLD and fibrosis pre- and post-COVID-19 pandemic

Firth regression analyzes revealed significant associations between sociodemographic, behavioral, and clinical factors and liver disease risk, using NHANES data from the pre-pandemic and pandemic periods. Interaction analyzes identified COVID-19 as a significant effect modifier across multiple associations. Given the large number of models and interaction tests performed, we acknowledge that some statistically significant findings may reflect chance. Accordingly, the results presented below emphasize associations supported by formal interaction terms and larger effect sizes, whereas isolated subgroup-specific estimates are reported but interpreted cautiously.

Socioeconomic indicators demonstrated heterogeneous interactions. Marriage or cohabitation consistently increased MASLD risk across periods (OR ≈ 1.5–1.6) (Table 3). Higher income predicted MetALD risk pre-pandemic (OR = 2.69), but this association was significantly attenuated after COVID-19 (ROR = 0.21) (Table 4). Employment status interacted with sex: unemployed women were more vulnerable to MetALD (ROR = 3.36), while full-time employment conferred protection (ROR = 0.11) (Supplementary Table S1). Educational attainment showed complex, sex-dependent associations with significant fibrosis. While having a college education or higher was protective among women during the pandemic (OR = 0.48; ROR = 0.12), risk-enhancing interaction was observed in men (OR = 1.48, ROR = 5.19; Supplementary Table S2).

**Table 3:** Associations between social and psychosocial determinants and liver disease pre- and post-COVID-19 pandemic.

Variables	Categories	MASLD		MetALD		ALD		Significant fibrosis	
		OR (95% CI) (2017–2020)	OR (95% CI) (2021–2023)	OR (95% CI) (2017–2020)	OR (95% CI) (2021–2023)	OR (95% CI) (2017–2020)	OR (95% CI) (2021–2023)	OR (95% CI) (2017–2020)	OR (95% CI) (2021–2023)
Educational levels	Less than college	Reference	Reference	Reference	Reference	Reference	Reference	Reference	Reference
	Some college	0.88 (0.65–1.18)	1.22 (0.79–1.89)	1.17 (0.56–2.44)	0.59 (0.36–0.95)	1.19 (0.50–2.81)	1.05 (0.29–3.83)	0.82 (0.48–1.39)	1.20 (0.76–1.88)
	College graduate or above	0.79 (0.59–1.07)	0.94 (0.59–1.49)	0.87 (0.46–1.62)	0.36 (0.17–0.79)	0.11 (0.04–0.29)*	0.32 (0.13–0.80)	0.45 (0.22–0.89)*	0.83 (0.50–1.38)
Married status	Never married	Reference	Reference	Reference	Reference	Reference	Reference	Reference	Reference
	Divorced, separated or widowed	1.09 (0.66–1.80)	1.62 (1.01–2.58)	0.65 (0.23–1.87)	1.09 (0.37–3.17)	1.55 (0.57–4.25)	1.53 (0.49–4.79)	0.75 (0.39–1.42)	0.84 (0.42–1.70)
	Married or living with partner	1.64 (1.15–2.33)*	1.47 (1.15–1.88)*	1.25 (0.55–2.87)	0.80 (0.30–2.16)	1.47 (0.56–3.87)	0.71 (0.24–2.08)	1.02 (0.55–1.88)	0.65 (0.35–1.20)
Place of birth	US-born	Reference	Reference	Reference	Reference	Reference	Reference	Reference	Reference
	Born outside the US	1.06 (0.75–1.49)	0.94 (0.51–1.74)	0.56 (0.31–1.02)	0.32 (0.12–0.87)	1.99 (0.97–4.05)	0.54 (0.18–1.65)	0.72 (0.47–1.10)	0.76 (0.43–1.37)
PIR levels	Low income	Reference	Reference	Reference	Reference	Reference	Reference	Reference	Reference
	Middle income	1.26 (0.88–1.79)	1.25 (0.73–2.15)	2.22 (1.08–4.55)*	0.51 (0.22–1.20)	0.76 (0.33–1.73)	0.73 (0.29–1.82)	0.99 (0.64–1.54)	1.42 (0.82–2.47)
	High income	1.23 (0.92–1.64)	1.09 (0.63–1.87)	2.69 (1.31–5.51)*	0.65 (0.28–1.54)	0.52 (0.23–1.16)	0.66 (0.19–2.31)	0.75 (0.44–1.30)	1.21 (0.66–2.23)
Work	Not-employed	Reference	Reference	Reference	Reference	Reference	Reference	Reference	Reference
	Part-time employee	1.24 (0.78–1.98)	0.93 (0.57–1.52)	0.80 (0.28–2.27)	0.71 (0.27–1.83)	0.66 (0.20–2.22)	1.58 (0.50–5.01)	1.93 (1.05–3.53)	1.29 (0.91–1.83)
	Full-time employee	1.56 (1.10–2.19)*	1.18 (0.94–1.49)	1.39 (0.70–2.76)	0.99 (0.62–1.60)	0.67 (0.24–1.91)	0.76 (0.32–1.78)	1.38 (0.92–2.09)	1.03 (0.79–1.35)
Health insurance	No	Reference	Reference	Reference	Reference	Reference	Reference	Reference	Reference
	Yes	1.23 (0.83–1.81)	0.76 (0.46–1.25)	0.85 (0.42–1.71)	1.41 (0.40–4.98)	0.32 (0.17–0.62)*	0.12 (0.05–0.27)*	0.67 (0.37–1.23)	1.26 (0.71–2.25)
Depression levels	No/minimal depression	Reference	Reference	Reference	Reference	Reference	Reference	Reference	Reference
	Depression-symptoms	0.83 (0.66–1.03)	1.06 (0.82–1.38)	2.18 (1.48–3.21)*	0.85 (0.44–1.64)	0.79 (0.33–1.92)	3.92 (1.91–8.02)*	0.62 (0.40–0.96)*	1.04 (0.68–1.59)
Smoking status	Never	Reference	Reference	Reference	Reference	Reference	Reference	Reference	Reference
	Ex-smoker	0.94 (0.77–1.15)	0.82 (0.64–1.05)	3.11 (1.14–8.52)*	2.22 (1.02–4.82)	1.67 (0.52–5.37)	6.83 (2.15–21.77)*	0.82 (0.53–1.27)	0.91 (0.65–1.28)
	Current smoker	0.73 (0.52–1.02)	1.07 (0.70–1.64)	7.15 (3.82–13.36)*	2.92 (1.59–5.35)*	3.49 (1.44–8.48)*	17.08 (6.45–45.19)*	0.95 (0.57–1.60)	1.05 (0.69–1.61)
PA levels	Low active	Reference	Reference	Reference	Reference	Reference	Reference	Reference	Reference
	Moderate active	1.09 (0.80–1.48)	1.09 (0.74–1.61)	0.74 (0.28–1.95)	1.24 (0.54–2.84)	0.66 (0.24–1.83)	0.40 (0.12–1.35)	0.78 (0.50–1.23)	1.16 (0.61–2.19)
	High active	0.71 (0.54–0.94)*	0.96 (0.68–1.34)	0.80 (0.42–1.52)	1.19 (0.61–2.34)	0.10 (0.05–0.22)*	1.32 (0.43–4.09)	0.71 (0.43–1.18)	1.16 (0.76–1.76)
Sleep hours (weekdays)	<6 h	Reference	Reference	Reference	Reference	Reference	Reference	Reference	Reference
	6-8 h	1.10 (0.66–1.85)	1.40 (1.03–1.91)	2.88 (1.19–6.97)*	0.84 (0.32–2.25)	0.88 (0.38–2.07)	1.06 (0.28–3.94)	0.92 (0.59–1.42)	0.80 (0.39–1.65)
	≥8 h	0.70 (0.41–1.18)	1.22 (0.94–1.59)	3.83 (1.21–12.15)*	0.89 (0.31–2.53)	0.42 (0.16–1.10)	1.25 (0.40–3.95)	0.80 (0.52–1.23)	0.85 (0.50–1.46)
Sleep hours (weekends)	<6 h	Reference	Reference	Reference	Reference	Reference	Reference	Reference	Reference
	6-8 h	1.02 (0.55–1.90)	0.86 (0.41–1.83)	1.81 (0.53–6.21)	0.84 (0.28–2.56)	0.28 (0.07–1.11)	0.18 (0.04–0.79)	0.97 (0.51–1.84)	0.68 (0.25–1.89)
	≥8 h	0.91 (0.51–1.64)	0.79 (0.35–1.79)	2.21 (0.82–5.97)	0.52 (0.17–1.63)	0.47 (0.17–1.30)	0.65 (0.22–1.91)	0.94 (0.50–1.76)	0.74 (0.33–1.66)
Energy intake, kcal		1.16 (1.04–1.29)*	1.11 (0.99–1.23)	1.43 (1.07–1.91)*	1.10 (0.77–1.56)	1.53 (1.26–1.85)*	0.97 (0.63–1.50)	1.16 (1.00–1.36)	1.33 (1.21–1.45)*
Alcohol consumption, g/week		0.48 (0.41–0.57)*	0.42 (0.37–0.49)*	2.01 (1.41–2.88)*	2.01 (1.72–2.34)*	2.61 (2.13–3.20)*	3.43 (2.38–4.97)*	1.14 (1.00–1.31)	1.14 (0.98–1.32)
CDAI		1.00 (0.89–1.14)	0.99 (0.90–1.10)	1.08 (0.79–1.47)	0.88 (0.63–1.23)	0.88 (0.61–1.27)	0.74 (0.35–1.56)	0.99 (0.79–1.23)	1.23 (1.06–1.43)*
DII		0.99 (0.86–1.14)	1.04 (0.89–1.21)	0.85 (0.64–1.14)	1.00 (0.73–1.37)	0.78 (0.54–1.14)	0.99 (0.65–1.49)	1.15 (0.91–1.47)	0.77 (0.68–0.89)*

Model was adjusted for age, sex, race, hypertension, diabetes mellitus and BMI. CI, confidence interval; ROR, ratio of odds ratios; MASLD, metabolic dysfunction-associated steatotic liver disease; MetALD, metabolic alcohol-related liver disease; ALD, alcohol-associated liver disease; PIR, poverty income ratio; CDAI, composite dietary antioxidant index; DII, dietary inflammatory index; PA, physical activity. ^*^p < 0.05.

**Table 4:** Significant interaction effects of social and psychosocial determinants with sex and pandemic period on liver diseases.

Disease	Interaction term	ROR (95% CI)	β (SE)	P-interaction
MASLD	Ex-smoker × men × pandemic	0.67 (0.48–0.94)	−0.40 (0.17)	0.030
	Alcohol consumption × depression symptoms × pandemic	0.41 (0.18–0.92)	−0.89 (0.41)	0.041
MetALD	Depression symptoms × pandemic	0.45 (0.21–0.96)	−0.80 (0.39)	0.048
	Middle income × pandemic	0.21 (0.06–0.70)	−1.56 (0.61)	0.017
	High income × pandemic	0.21 (0.07–0.69)	−1.54 (0.60)	0.016
	Depression symptoms × women × pandemic	0.09 (0.01–0.86)	−2.37 (1.14)	0.047
	High income × men × pandemic	0.16 (0.03–0.81)	−1.85 (0.84)	0.038
	Not-employed × women × pandemic	3.36 (1.08–10.46)	1.21 (0.58)	0.048
	Full-time employee × women × pandemic	0.11 (0.02–0.47)	−2.24 (0.76)	0.008
	Alcohol consumption × women × pre-pandemic	3.10 (1.60–5.99)	1.13 (0.34)	0.002
	Alcohol consumption × women × pandemic	0.43 (0.21–0.87)	−0.85 (0.37)	0.027
	Current smoker × depression symptoms × pre-pandemic	0.20 (0.05–0.87)	−1.60 (0.75)	0.044
	Current smoker × pandemic	0.20 (0.08–0.47)	−1.61 (0.44)	0.001
	Never-smoker × depression symptoms × pandemic	0.21 (0.05–0.84)	−1.57 (0.71)	0.038
ALD	Born outside the US × pandemic	0.06 (0.01–0.61)	−2.81 (1.18)	0.025
	Depression symptoms × men × pandemic	6.73 (1.50–30.13)	1.91 (0.76)	0.019
	Sleep hours (weekends) ≥ 8 h × women × pre-pandemic	0.09 (0.01–0.75)	−2.37 (1.06)	0.037
Significant Fibrosis	College graduate or above × women × pre-pandemic	3.01 (1.24–7.27)	1.10 (0.45)	0.023
	Some college × men × pandemic	2.65 (1.09–6.42)	0.97 (0.45)	0.042
	College graduate or above × men × pandemic	5.19 (2.05–13.11)	1.65 (0.47)	0.002
	Less than college × women × pandemic	3.92 (1.80–8.51)	1.37 (0.40)	0.002
	College graduate or above × women × pandemic	0.12 (0.03–0.39)	−2.15 (0.62)	0.002
	Current smoker × women × pandemic	3.42 (1.07–10.89)	1.23 (0.59)	0.049
	Sleep hours (weekends) ≥ 8 h × women × pandemic	0.10 (0.01–0.65)	−2.33 (0.96)	0.025
	DII × men × pandemic	0.61 (0.43–0.86)	−0.50 (0.18)	0.009
	Some college × divorced, separated or widowed × pandemic	0.12 (0.02–0.72)	−2.16 (0.93)	0.035

Model was adjusted for age, sex, race, hypertension, diabetes mellitus and BMI. Reference groups: pre-pandemic period, less than college, never married, men, never-smoker, US-born, low income, not employed, no/minimal depression, no health insurance, low active, and 6–8 h sleep. NA indicates not applicable for continuous × categorical interactions. ROR, ratio of odds ratios; MASLD, metabolic dysfunction-associated steatotic liver disease; MetALD, metabolic alcohol-related liver disease; ALD, alcohol-associated liver disease; DII, dietary inflammatory index.

Psychosocial factors revealed striking effect modifications. In MetALD, depression was a strong risk factor pre-pandemic (OR = 2.18), particularly among women (OR = 4.57), but this association was abolished during the pandemic (overall OR = 0.85, ROR = 0.45; women OR = 0.44, ROR = 0.09). In ALD, depressive symptoms notably increased the risk, particularly among men during the pandemic. In the overall population, the association was non-significant pre-pandemic (OR = 0.79) but became significant post-pandemic (OR = 3.92). Stratified analysis by sex revealed that this shift was driven predominantly by men, in whom depressive symptoms were not a risk factor pre-pandemic (OR = 1.03) but became a strong predictor during the pandemic (OR = 5.31). In contrast, the association remained non-significant in women in both periods. The formal three-way interaction (depressive symptoms × men × pandemic period) was statistically significant (ROR = 6.73, *p* = 0.019), confirming a sex-specific effect modification. For MASLD, depressive symptoms alone were not significant predictors; however, a notable three-way interaction emerged, showing that the joint effect of alcohol consumption and depressive symptoms on MASLD risk was significantly attenuated during the pandemic (ROR = 0.41, *p* = 0.041).

Behavioral determinants showed distinct and pandemic-modified effects across liver disease phenotypes. Alcohol consumption consistently exhibited an inverse association with MASLD both before and during the pandemic (OR = 0.48 pre-pandemic; OR = 0.42 pandemic), whereas it strongly predicted MetALD (OR = 2.01 in both periods) and ALD (OR rising from 2.61 to 3.43). Current smoking similarly intensified ALD risk (post-pandemic OR = 17.08 vs. 3.49 pre-pandemic), while its association with MetALD attenuated (OR = 7.15 to 2.92; ROR = 0.20). Physical activity provided robust protection against ALD prior to the pandemic (OR = 0.10 overall; OR = 0.04 in men), but these effects were abolished during the pandemic (OR = 1.32 overall). For MASLD, high physical activity was protective in men before COVID-19 (OR = 0.58) but lost significance thereafter. Notably, former smoking emerged as a pandemic-specific protective factor for MASLD among men (OR = 0.65), suggesting behavioral shifts modified disease vulnerability.

Dietary and lifestyle factors further contributed to fibrosis risk. During the pandemic, higher energy intake predicted significant fibrosis (OR = 1.33), whereas a higher DII conferred protection (OR = 0.77). Importantly, the pro-inflammatory effect of diet was attenuated post-pandemic (ROR = 0.61). Sleep patterns also emerged as significant modifiers. During the pandemic, longer weekend sleep duration was associated with a significantly reduced risk of fibrosis among women. Specifically, compared to those sleeping less than 6 h, women who slept 6–8 h on weekends had a 70% lower risk (OR = 0.30), and those sleeping ≥8 h had a 65% lower risk (OR = 0.35). This graded, protective association remained significant after comprehensive adjustment for age, race, depressive symptoms, energy intake, and dietary indices (CDAI and DII; Supplementary Table S3).

## 4 Discussion

In this nationally representative repeated cross-sectional analysis of NHANES data from 2017 to 2023, we observed that the prevalence of SLD phenotypes and the strength of their associations with social, psychosocial, and behavioral factors differed between the pre-pandemic and pandemic periods In this nationally representative repeated cross-sectional analysis of NHANES data from 2017 to 2023, we observed that the prevalence of SLD phenotypes and the strength of their associations with social, psychosocial, and behavioral factors differed between the pre-pandemic and pandemic periods. While we observed a modest decline in the prevalence of MASLD after the pandemic, the burden of significant fibrosis increased, particularly among women. This divergence presents a critical paradox: while overt fatty liver disease appeared less common, the severity of fibrosis — a key driver of morbidity and mortality — worsened in specific subgroups. These shifts suggest that pandemic-related disruptions in social and behavioral factors may have differentially influenced disease onset and progression, underscoring the need to move beyond simple prevalence estimates toward a more nuanced understanding of disease heterogeneity.

Socioeconomic factors exhibited highly dynamic roles throughout the pandemic. Higher income appeared to mitigate the risk of MetALD during the pandemic, suggesting that financial stability helped buffer individuals from psychosocial and behavioral stressors that could lead to harmful drinking and increased metabolic risk (20). Full-time employment, which reduced risk of MetALD among women, may provide financial security, social status, and personal development, which buffer against psychosocial stressors (21). The sex-specific protective role of education against fibrosis progression during the pandemic further underscores the complexity of social gradients. While higher education shielded women from fibrosis progression, it paradoxically increased the risk for men. These findings suggest that education functions not only as a socioeconomic indicator but also as a modifier of coping strategies and lifestyle choices in times of social upheaval. Previous studies have noted that socioeconomic gradients in liver fibrosis vary by sex and ethnicity (22, 23), but our results emphasize how these gradients can invert or diminish under societal shocks, revealing that they are contingent rather than fixed.

Loneliness and depressive symptoms have been found to mediate the relationship between alcohol use disorder (AUD) and psychological distress during the pandemic (24). We found that depressive symptoms emerged as a significant risk factor for ALD during the pandemic, particularly among men. This suggests that pandemic-related psychosocial stress, isolation, and disruptions to coping mechanisms may have exacerbated maladaptive alcohol use in men, leading to direct hepatic injury and worsening of ALD (25). Conversely, the previously strong association between depressive symptoms and MetALD in women disappeared post-pandemic, suggesting either increased resilience or shifts in alcohol consumption patterns among women facing heightened stress. The three-way interaction in MASLD, where the combined effect of alcohol use and depressive symptoms was reduced post-pandemic, suggests that disruptions in social drinking contexts or heightened health awareness may have altered risk profiles. While alcohol consumption remained inversely associated with MASLD, it consistently heightened the risk of MetALD and ALD, with the risk for ALD becoming more pronounced post-pandemic. This bifurcation underscores the importance of differentiating between metabolic- and alcohol-related pathways, as aggregated alcohol metrics mask fundamentally distinct mechanisms (26).

A web-based survey revealed that smoking increased during the COVID-19 pandemic, particularly among vulnerable populations, with stress, anxiety, and unemployment contributing to higher cigarette consumption (27). Smoking accelerates liver disease progression by promoting lipid accumulation and fibrosis through mechanisms such as oxidative stress and increased inflammatory cytokines (28, 29). We found that current smoking significantly increased the risk of ALD during the pandemic, while its association with MetALD weakened. Interestingly, former smoking appeared to serve as a protective factor against MASLD in men. These temporal and sex-specific shifts may reflect pandemic-driven changes in nicotine use behaviors, cessation efforts, and broader lifestyle adaptations. Physical activity, which was previously considered a strong protective factor against ALD and MASLD, lost its significance during the pandemic. This shift suggests that the protective effects of physical activity may have been attenuated by structural barriers to consistent engagement during the pandemic. This suggests that disruptions in the quality, intensity, or consistency of activity patterns may have occurred, even if overall activity levels improved. This is consistent with emerging studies indicating that pandemic-related disruptions affected activity patterns, with reductions in occupational and structured exercise potentially offsetting increases in leisure-time or home-based activity (30, 31).

Dietary and lifestyle factors also played a significant role in shaping the risk of fibrosis. Contrary to expectations, higher DII scores were inversely associated with significant fibrosis, with the strength of this protective effect increasing during the pandemic. Although counterintuitive, this finding may reflect confounding factors such as underreporting of unhealthy diets, shifts in food insecurity, or survival bias, and warrants further investigation in independent cohorts. Sleep duration also acted as a modifier, with longer weekend sleep serving as a protective factor against fibrosis in women. Several studies have shown that sleep deprivation and poor sleep quality increase oxidative stress and promote a pro-inflammatory state, which in turn contributes to insulin resistance and metabolic risk (32–34). Adequate sleep, on the other hand, reduces systemic inflammation and improves insulin sensitivity, helping to mitigate hepatic stress.

Overall, our findings suggest that associations between psychosocial and behavioral factors and SLD and fibrosis differed across pandemic periods and were not uniform across sexes or contexts. The distinct patterns observed for MASLD, MetALD, and ALD across survey periods, together with the higher prevalence of fibrosis observed during the pandemic period, highlight the potential value of incorporating social, psychological, and behavioral factors into liver disease risk models. In clinical and public health practice, these results advocate for precision prevention strategies that account for shifting risk profiles under societal stressors. Importantly, they also suggest that crises such as the COVID-19 pandemic can serve as natural experiments, revealing latent vulnerabilities and protective factors that are typically obscured in stable contexts.

Several limitations of our study should be considered. First, the repeated cross-sectional design of NHANES limits the ability to establish causal relationships between the pandemic period, psychosocial factors, and SLD outcomes. Future prospective studies with repeated vibration-controlled transient elastography (VCTE) measurements are needed to assess temporal relationships and elucidate potential mechanisms. Second, self-reported data on diet, physical activity, alcohol consumption, and psychosocial factors are subject to recall and social desirability biases, which may have led to exposure misclassification. Third, NHANES data collection during 2021–2023 occurred under pandemic-related constraints and was associated with lower response rates compared with pre-pandemic cycles. Although NHANES sampling weights incorporate adjustments for non-response, residual selection bias due to unmeasured differences between respondents and non-respondents during the pandemic period cannot be excluded, potentially affecting the generalizability of pandemic-period estimates. Fourth, the large number of statistical tests performed across multiple outcomes, determinants, and interaction models substantially increases the risk of Type I error. While our analyses were hypothesis-driven and focused on effect modification by pandemic period and sex, and while we prioritized interpretation of interaction patterns, effect sizes, and findings consistent with prior biological knowledge, many individual associations—particularly within subgroup analyses—may reflect chance findings and require independent replication. Fifth, although VCTE provides a validated and widely used noninvasive assessment of hepatic steatosis and fibrosis, some degree of misclassification relative to liver biopsy remains possible. Finally, residual confounding from unmeasured factors, such as pandemic-related changes in medication use, undocumented SARS-CoV-2 infections, or post-COVID syndrome, which may influence metabolic health and liver injury, cannot be excluded.

## 5 Conclusion

The COVID-19 pandemic period provided a unique context in which the associations of psychosocial and behavioral factors with liver disease differed in complex, sex-specific ways. The paradoxical decline in MASLD, alongside worsening fibrosis, underscores the limitations of prevalence metrics and advocates for a shift toward dynamic models of disease progression. Future prevention and intervention strategies should integrate mental health, lifestyle modifications, and structural supports, with explicit attention to gendered vulnerabilities. Implementing such an integrated framework will be crucial in mitigating the post-pandemic burden of SLD and addressing long-term social determinants of liver health.

## Data Availability

The original contributions presented in the study are included in the article/Supplementary material, further inquiries can be directed to the corresponding author.

## Glossary

ALD: Alcohol-associated liver disease
AUD: Alcohol use disorder
BMI: Body mass index
CAP: Controlled Attenuation Parameter
CDAI: Composite Dietary Antioxidant Index
CMRFs: Cardiometabolic risk factors
DBP: Diastolic blood pressure
DII: Dietary Inflammatory Index
HDL-C: High-density lipoprotein cholesterol
HRQOL: Health-related quality of life
LSM: Liver stiffness measurement
MASLD: Metabolic dysfunction-associated steatotic liver disease
MetALD: Metabolic alcohol-related liver disease
NHANES: National Health and Nutrition Examination Survey
OR: Odds ratio
PA: Physical activity
PIR: Poverty-income ratio
ROR: Ratio of odds ratios
SBP: Systolic blood pressure
SLD: Steatotic liver disease
TC: Total cholesterol
VCTE: Vibration-controlled transient elastography
WC: Waist circumference
WHO: World Health Organization
