# Supplementary material for: The impact of the COVID-19 pandemic on liver health: exploring shifts in social and psychosocial determinants of steatosis and fibrosis
Source: Front Med (Lausanne). 2026 Feb 3;13:1725338. doi: 10.3389/fmed.2026.1725338 (PMC12909494; doi:10.3389/fmed.2026.1725338)
Supplement: Supplementary file 1 [file Data_Sheet_1.docx]

**Figure. S1.** Flowchart for selection of study population


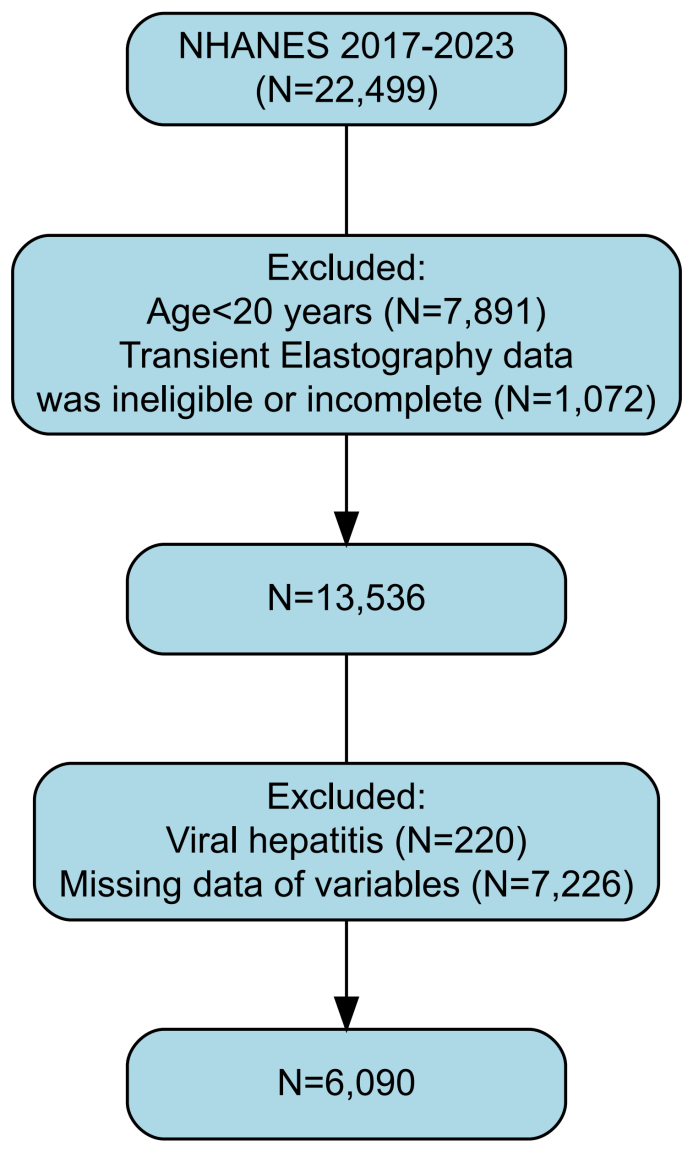


**Table S1.** Associations between social and psychosocial determinants and liver disease among women pre- and post-COVID-19 pandemic.

| Variables |  | MASLD | | MetALD | | ALD | | Significant fibrosis | |
| --- | --- | --- | --- | --- | --- | --- | --- | --- | --- |
|  |  | OR （95%CI）(2017-2020) | OR （95%CI）(2021-2023) | OR （95%CI）(2017-2020) | OR （95%CI）(2021-2023) | OR （95%CI）(2017-2020) | OR （95%CI）(2021-2023) | OR （95%CI）(2017-2020) | OR （95%CI）(2021-2023) |
| Educational levels | Less than college | Reference | Reference | Reference | Reference | Reference | Reference | Reference | Reference |
|  | Some college | 0.78 (0.53-1.15) | 1.00 (0.53-1.88) | 0.65 (0.18-2.36) | 0.53 (0.20-1.40) | 1.19 (0.61-2.29) | 0.87 (0.23-3.33) | 1.14 (0.50-2.60) | 0.81 (0.47-1.41) |
|  | College graduate or above | 0.70 (0.44-1.13) | 0.74 (0.38-1.43) | 0.79 (0.24-2.59) | 0.41 (0.16-1.06) | 0.81 (0.49-1.35) | 0.71 (0.20-2.52) | 0.88 (0.33-2.37) | 0.48 (0.26-0.92) |
| Married status | Never married | Reference | Reference | Reference | Reference | Reference | Reference | Reference | Reference |
|  | Divorced, separated or widowed | 1.18 (0.69-2.02) | 1.77 (0.85-3.67) | 0.99 (0.18-5.53) | 1.17 (0.25-5.42) | 0.50 (0.26-0.98) | 1.32 (0.42-4.13) | 0.55 (0.17-1.77) | 1.29 (0.55-2.98) |
|  | Married or living with partner | 1.22 (0.77-1.92) | 1.62 (0.95-2.76) | 1.48 (0.35-6.32) | 0.93 (0.34-2.55) | 0.46 (0.23-0.95) | 0.22 (0.12-0.43)* | 0.63 (0.20-1.94) | 0.78 (0.42-1.45) |
| Place of birth | US-born | Reference | Reference | Reference | Reference | Reference | Reference | Reference | Reference |
|  | Born outside the US | 1.00 (0.56-1.78) | 0.69 (0.26-1.80) | 0.29 (0.12-0.66)* | 0.07 (0.02-0.20)* | 0.94 (0.34-2.57) | 0.73 (0.46-1.14) | 0.80 (0.48-1.35) | 0.32 (0.17-0.62)* |
| PIR levels | Low income | Reference | Reference | Reference | Reference | Reference | Reference | Reference | Reference |
|  | Middle income | 1.28 (0.81-2.03) | 1.29 (0.70-2.38) | 1.52 (0.58-3.99) | 0.29 (0.11-0.73) | 1.78 (0.88-3.60) | 0.23 (0.06-0.82) | 0.95 (0.53-1.69) | 1.05 (0.48-2.27) |
|  | High income | 1.18 (0.77-1.81) | 1.02 (0.52-1.97) | 2.19 (0.85-5.69) | 0.71 (0.27-1.85) | 0.56 (0.36-0.86)* | 0.20 (0.06-0.61)* | 0.65 (0.36-1.20) | 0.70 (0.29-1.72) |
| Work | Not-employed | Reference | Reference | Reference | Reference | Reference | Reference | Reference | Reference |
|  | Part-time employee | 1.25 (0.69-2.26) | 0.89 (0.49-1.62) | 1.32 (0.32-5.38) | 0.35 (0.11-1.14) | 0.97 (0.54-1.77) | 0.96 (0.34-2.68) | 1.64 (0.65-4.14) | 0.63 (0.29-1.39) |
|  | Full-time employee | 1.55 (0.96-2.48) | 1.04 (0.76-1.42) | 1.35 (0.35-5.17) | 0.32 (0.14-0.74) | 0.29 (0.17-0.52)* | 1.00 (0.54-1.85) | 1.21 (0.59-2.47) | 0.91 (0.70-1.20) |
| Health insurance | No | Reference | Reference | Reference | Reference | Reference | Reference | Reference | Reference |
|  | Yes | 0.91 (0.47-1.78) | 0.64 (0.27-1.51) | 0.44 (0.15-1.29) | 0.63 (0.13-3.15) | 1.18 (0.52-2.69) | 0.52 (0.27-1.00) | 0.73 (0.35-1.49) | 2.54 (0.78-8.24) |
| Depression levels | No/minimal depression | Reference | Reference | Reference | Reference | Reference | Reference | Reference | Reference |
|  | Depression-symptoms | 0.90 (0.67-1.22) | 1.14 (0.80-1.62) | 4.57 (1.98-10.51)* | 0.44 (0.17-1.12) | 1.18 (0.62-2.24) | 2.45 (0.81-7.45) | 0.94 (0.49-1.82) | 0.98 (0.56-1.69) |
| Smoking status | Never | Reference | Reference | Reference | Reference | Reference | Reference | Reference | Reference |
|  | Ex-smoker | 0.75 (0.49-1.16) | 1.04 (0.64-1.71) | 2.23 (0.90-5.53) | 1.18 (0.55-2.51) | 2.03 (0.98-4.21) | 9.69 (4.04-23.23)* | 0.85 (0.40-1.82) | 1.76 (1.20-2.57)* |
|  | Current smoker | 1.02 (0.62-1.68) | 1.62 (0.95-2.77) | 10.65 (2.92-38.85)* | 2.83 (0.98-8.14) | 0.91 (0.38-2.15) | 11.39 (3.98-32.63)* | 0.94 (0.45-1.96) | 2.17 (1.23-3.83) |
| PA levels | Low active | Reference | Reference | Reference | Reference | Reference | Reference | Reference | Reference |
|  | Moderate active | 1.22 (0.74-2.01) | 1.17 (0.79-1.73) | 0.34 (0.10-1.13) | 1.45 (0.70-3.02) | 2.74 (1.61-4.66)* | 1.08 (0.42-2.82) | 1.20 (0.61-2.38) | 1.26 (0.56-2.84) |
|  | High active | 0.74 (0.40-1.39) | 0.86 (0.57-1.30) | 0.77 (0.27-2.17) | 0.74 (0.32-1.70) | 1.94 (1.07-3.53)* | 3.05 (1.99-4.68)* | 0.50 (0.26-0.98) | 1.45 (0.85-2.49) |
| Sleep hours (weekdays) | <6h | Reference | Reference | Reference | Reference | Reference | Reference | Reference | Reference |
|  | 6-8h | 0.91 (0.34-2.42) | 1.50 (0.72-3.15) | 4.77 (1.81-12.53)* | 0.93 (0.32-2.70) | 0.21 (0.13-0.33)* | 0.46 (0.17-1.20) | 0.70 (0.32-1.55) | 0.58 (0.27-1.26) |
|  | ≥8h | 0.65 (0.27-1.56) | 1.44 (0.81-2.55) | 7.43 (3.06-18.01)* | 1.23 (0.40-3.78) | 0.06 (0.03-0.13)* | 0.81 (0.51-1.29) | 0.61 (0.27-1.38) | 0.61 (0.32-1.19) |
| Sleep hours (weekends) | <6h | Reference | Reference | Reference | Reference | Reference | Reference | Reference | Reference |
|  | 6-8h | 0.86 (0.28-2.64) | 1.03 (0.38-2.78) | 1.19 (0.20-7.06) | 2.59 (1.26-5.30) | 0.17 (0.10-0.29)* | 0.13 (0.07-0.23)* | 1.86 (0.54-6.37) | 0.30 (0.16-0.57)* |
|  | ≥8h | 0.93 (0.33-2.57) | 0.99 (0.32-3.07) | 1.72 (0.43-6.90) | 1.61 (1.02-2.54) | 0.12 (0.06-0.24)* | 0.49 (0.25-0.95) | 1.85 (0.65-5.26) | 0.35 (0.19-0.67)* |
| Energy intake, kcal |  | 1.05 (0.87-1.26) | 0.98 (0.79-1.23) | 1.73 (1.21-2.46)* | 0.75 (0.34-1.66) | 1.67 (1.24-2.26)* | 0.40 (0.12-1.37) | 1.09 (0.83-1.44) | 1.02 (0.68-1.55) |
| Alcohol consumption, g/week |  | 0.38 (0.24-0.59)* | 0.30 (0.17-0.52)* | 5.38 (2.47-11.72)* | 2.40 (1.74-3.30)* | 2.58 (2.12-3.13)* | 6.64 (4.23-10.43)* | 0.66 (0.31-1.37) | 1.25 (0.97-1.61) |
| CDAI |  | 0.93 (0.80-1.08) | 0.86 (0.70-1.05) | 1.16 (0.77-1.75) | 0.94 (0.49-1.79) | 1.52 (1.26-1.83)* | 0.38 (0.10-1.40) | 1.01 (0.75-1.36) | 1.23 (0.93-1.62) |
| DII |  | 1.10 (0.91-1.32) | 1.26 (1.03-1.53) | 0.78 (0.49-1.24) | 0.86 (0.55-1.34) | 1.04 (0.86-1.26) | 1.92 (0.93-3.97) | 1.24 (0.92-1.68) | 0.84 (0.66-1.05) |

Model was adjusted for age, race, hypertension, diabetes mellitus and BMI. CI, confidence interval; ROR, ratio of odds ratios; MASLD: metabolic dysfunction-associated steatotic liver disease; MetALD: metabolic alcohol-related liver disease; ALD: alcohol-associated liver disease; PIR: poverty income ratio; CDAI: composite dietary antioxidant index; DII: dietary inflammatory index; PA, physical activity. ^*^P<0.05.

**Table S2.** Associations between social and psychosocial determinants and liver disease among men pre- and post-COVID-19 pandemic.

| Variables |  | MASLD | | MetALD | | ALD | | Significant fibrosis | |
| --- | --- | --- | --- | --- | --- | --- | --- | --- | --- |
|  |  | OR （95%CI）(2017-2020) | OR （95%CI）(2021-2023) | OR （95%CI）(2017-2020) | OR （95%CI）(2021-2023) | OR （95%CI）(2017-2020) | OR （95%CI）(2021-2023) | OR （95%CI）(2017-2020) | OR （95%CI）(2021-2023) |
| Educational levels | Less than college | Reference | Reference | Reference | Reference | Reference | Reference | Reference | Reference |
|  | Some college | 1.00 (0.58-1.72) | 1.49 (0.85-2.61) | 1.80 (0.70-4.61) | 0.75 (0.30-1.83) | 1.30 (0.53-3.18) | 1.15 (0.33-4.00) | 0.71 (0.37-1.38) | 1.91 (1.01-3.60) |
|  | College graduate or above | 0.89 (0.61-1.29) | 1.20 (0.73-1.95) | 1.05 (0.62-1.79) | 0.41 (0.14-1.21) | 0.12 (0.04-0.34)* | 0.21 (0.07-0.60)* | 0.30 (0.14-0.63)* | 1.48 (0.76-2.85) |
| Married status | Never married | Reference | Reference | Reference | Reference | Reference | Reference | Reference | Reference |
|  | Divorced, separated or widowed | 0.97 (0.48-1.97) | 1.76 (0.98-3.17) | 0.31 (0.10-0.91) | 1.07 (0.30-3.75) | 2.80 (0.85-9.15) | 1.50 (0.38-5.98) | 0.86 (0.26-2.86) | 0.51 (0.16-1.61) |
|  | Married or living with partner | 1.92 (1.12-3.30)* | 1.36 (0.87-2.13) | 1.05 (0.31-3.50) | 0.75 (0.23-2.41) | 1.67 (0.49-5.75) | 0.91 (0.31-2.69) | 1.16 (0.41-3.33) | 0.52 (0.22-1.21) |
| Place of birth | US-born | Reference | Reference | Reference | Reference | Reference | Reference | Reference | Reference |
|  | Born outside the US | 1.17 (0.82-1.67) | 1.16 (0.60-2.27) | 0.74 (0.36-1.51) | 0.82 (0.36-1.89) | 2.10 (1.06-4.17)* | 0.53 (0.15-1.89) | 0.70 (0.34-1.43) | 1.19 (0.40-3.57) |
| PIR levels | Low income | Reference | Reference | Reference | Reference | Reference | Reference | Reference | Reference |
|  | Middle income | 1.18 (0.75-1.84) | 1.12 (0.45-2.76) | 2.37 (0.96-5.85) | 0.49 (0.15-1.64) | 0.71 (0.27-1.86) | 1.25 (0.44-3.53) | 0.90 (0.47-1.74) | 2.03 (0.71-5.81) |
|  | High income | 1.07 (0.74-1.55) | 0.99 (0.47-2.11) | 2.51 (0.96-6.56) | 0.42 (0.16-1.08) | 0.47 (0.19-1.14) | 1.00 (0.31-3.17) | 0.68 (0.32-1.42) | 2.02 (0.70-5.81) |
| Work | Not-employed | Reference | Reference | Reference | Reference | Reference | Reference | Reference | Reference |
|  | Part-time employee | 1.41 (0.71-2.81) | 0.95 (0.49-1.84) | 0.44 (0.13-1.51) | 2.12 (0.49-9.18) | 0.77 (0.21-2.86) | 2.51 (0.67-9.41) | 2.44 (1.13-5.26)* | 2.64 (1.61-4.35)* |
|  | Full-time employee | 1.35 (0.83-2.19) | 1.15 (0.83-1.60) | 1.10 (0.55-2.19) | 2.13 (1.19-3.81) | 0.51 (0.18-1.45) | 0.63 (0.21-1.87) | 1.24 (0.75-2.02) | 1.18 (0.76-1.81) |
| Health insurance | No | Reference | Reference | Reference | Reference | Reference | Reference | Reference | Reference |
|  | Yes | 1.64 (1.07-2.51)* | 0.82 (0.46-1.45) | 1.17 (0.55-2.49) | 1.82 (0.43-7.69) | 0.30 (0.16-0.57)* | 0.11 (0.04-0.30)* | 0.73 (0.33-1.61) | 0.93 (0.39-2.26) |
| Depression levels | No/minimal depression | Reference | Reference | Reference | Reference | Reference | Reference | Reference | Reference |
|  | Depression-symptoms | 0.88 (0.64-1.23) | 1.16 (0.79-1.72) | 1.65 (0.96-2.82) | 1.31 (0.47-3.68) | 1.03 (0.41-2.58) | 5.31 (2.64-10.68)* | 0.48 (0.26-0.91)* | 1.14 (0.59-2.20) |
| Smoking status | Never | Reference | Reference | Reference | Reference | Reference | Reference | Reference | Reference |
|  | Ex-smoker | 0.94 (0.76-1.17) | 0.65 (0.49-0.86)* | 3.29 (1.26-8.59)* | 3.30 (1.61-6.74)* | 1.36 (0.42-4.43) | 5.30 (1.25-22.47) | 0.74 (0.46-1.19) | 0.52 (0.29-0.93) |
|  | Current smoker | 0.53 (0.31-0.92)* | 0.85 (0.48-1.50) | 4.60 (2.67-7.94)* | 2.75 (1.21-6.23) | 3.58 (1.45-8.84)* | 18.71 (5.31-65.96)* | 1.04 (0.57-1.92) | 0.60 (0.32-1.14) |
| PA levels | Low active | Reference | Reference | Reference | Reference | Reference | Reference | Reference | Reference |
|  | Moderate active | 0.94 (0.55-1.58) | 0.94 (0.58-1.54) | 1.09 (0.37-3.18) | 1.26 (0.43-3.73) | 0.59 (0.20-1.74) | 0.33 (0.09-1.17) | 0.55 (0.28-1.07) | 1.03 (0.45-2.34) |
|  | High active | 0.58 (0.41-0.82)* | 0.88 (0.60-1.30) | 0.74 (0.29-1.89) | 1.17 (0.43-3.16) | 0.04 (0.03-0.07)* | 0.73 (0.19-2.84) | 0.69 (0.40-1.18) | 0.82 (0.51-1.32) |
| Sleep hours (weekdays) | <6h | Reference | Reference | Reference | Reference | Reference | Reference | Reference | Reference |
|  | 6-8h | 1.25 (0.57-2.75) | 1.34 (0.91-1.95) | 1.99 (0.88-4.52) | 0.91 (0.28-2.94) | 1.17 (0.48-2.86) | 0.99 (0.30-3.28) | 1.04 (0.65-1.69) | 0.92 (0.34-2.48) |
|  | ≥8h | 0.80 (0.37-1.76) | 1.11 (0.78-1.60) | 2.90 (0.91-9.24) | 0.88 (0.22-3.50) | 0.72 (0.27-1.94) | 1.13 (0.39-3.25) | 1.13 (0.65-1.96) | 1.03 (0.47-2.24) |
| Sleep hours (weekends) | <6h | Reference | Reference | Reference | Reference | Reference | Reference | Reference | Reference |
|  | 6-8h | 1.11 (0.58-2.12) | 0.71 (0.28-1.83) | 1.92 (0.56-6.59) | 0.45 (0.13-1.61) | 0.34 (0.08-1.50) | 0.16 (0.03-0.78) | 0.65 (0.29-1.43) | 1.19 (0.19-7.49) |
|  | ≥8h | 0.97 (0.54-1.76) | 0.65 (0.23-1.85) | 2.41 (0.85-6.87) | 0.31 (0.10-1.03) | 0.69 (0.24-2.00) | 0.59 (0.20-1.74) | 0.70 (0.35-1.39) | 1.29 (0.34-4.93) |
| Energy intake, kcal |  | 1.06 (0.95-1.19) | 1.04 (0.89-1.22) | 1.28 (0.91-1.81) | 1.09 (0.74-1.61) | 1.34 (1.05-1.70)* | 0.85 (0.52-1.38) | 1.04 (0.87-1.24) | 1.39 (1.18-1.65)* |
| Alcohol consumption, g/week |  | 0.38 (0.32-0.47)* | 0.36 (0.27-0.48)* | 1.74 (1.38-2.20)* | 1.81 (1.53-2.14)* | 2.46 (2.01-3.01)* | 2.88 (1.73-4.79)* | 1.13 (0.97-1.30) | 1.08 (0.93-1.26) |
| CDAI |  | 1.05 (0.88-1.27) | 1.07 (0.94-1.21) | 1.04 (0.65-1.68) | 0.89 (0.58-1.36) | 0.85 (0.55-1.31) | 0.86 (0.49-1.50) | 0.96 (0.76-1.20) | 1.20 (0.94-1.54) |
| DII |  | 1.03 (0.83-1.27) | 0.97 (0.79-1.19) | 0.99 (0.60-1.62) | 1.15 (0.76-1.73) | 0.88 (0.58-1.32) | 0.96 (0.63-1.44) | 1.25 (0.96-1.62) | 0.77 (0.61-0.97) |

Model was adjusted for age, race, hypertension, diabetes mellitus and BMI. CI, confidence interval; ROR, ratio of odds ratios; MASLD: metabolic dysfunction-associated steatotic liver disease; MetALD: metabolic alcohol-related liver disease; ALD: alcohol-associated liver disease; PIR: poverty income ratio; CDAI: composite dietary antioxidant index; DII: dietary inflammatory index; PA, physical activity. ^*^P<0.05.
